# Identification of circulating miRNA biomarkers based on global quantitative real-time PCR profiling

**DOI:** 10.1186/2049-1891-3-4

**Published:** 2012-02-28

**Authors:** Kang Kang, Xiao Peng, Jun Luo, Deming Gou

**Affiliations:** 1 College of Life Science, Shenzhen University, Shenzhen, Guangdong, 518060, China; 2 College of Animal Science and Technology, Northwest A&F University, Yangling, Shaanxi, 712100, China

**Keywords:** biomarker, circulating microRNAs, profiling, quantitative real-time PCR

## Abstract

MicroRNAs (miRNAs) are small noncoding RNAs (18-25 nucleotides) that regulate gene expression at the post-transcriptional level. Recent studies have demonstrated the presence of miRNAs in the blood circulation. Deregulation of miRNAs in serum or plasma has been associated with many diseases including cancers and cardiovascular diseases, suggesting the possible use of miRNAs as diagnostic biomarkers. However, the detection of the small amount of miRNAs found in serum or plasma requires a method with high sensitivity and accuracy. Therefore, the current study describes polymerase chain reaction (PCR)-based methods for measuring circulating miRNAs. Briefly, the procedure involves four major steps: (1) sample collection and preparation; (2) global miRNAs profiling using quantitative real-time PCR (qRT-PCR); (3) data normalization and analysis; and (4) selection and validation of miRNA biomarkers. In conclusion, qRT-PCR is a promising method for profiling of circulating miRNAs as biomarkers.

## Background

MicroRNAs (miRNAs), a class of 18 to 25 noncoding nucleotides, are capable of regulating gene expression through messenger RNA degradation or translational repression and are involved in various biological processes, such as proliferation, differentiation, development, and apoptosis [1,2]. Recently, the presence of miRNAs in the blood circulation has been reported [3]. Interestingly, deregulation of circulating miRNAs has been associated with a variety of human diseases, including cancer [4,5] and cardiovascular diseases [6,7], indicating that miRNAs could be used as biomarkers for cancer and other diseases.

Several methods, such as northern blot [8], bead-based flow cytometry [9], microarray [10,11], quantitative real-time PCR (qRT-PCR) [12-14], and deep sequencing [15,16] have been developed to measure miRNA expression [17]. Of these methods, qRT-PCR is superior due to its high sensitivity, specificity and reproducibility. While other methods, such as microarray, require a larger amount of RNA sample (usually more than 1 μg), qRT-PCR requires less RNA input, where even as little as a single cell can be used for profiling [18,19]. Since the expression levels of circulating miRNAs are very low, qRT-PCR is well adapted for analyzing circulating miRNAs profiles because of its sensitivity. In addition, approximately 1,900 mature miRNAs have been found in human genome (miRbase 18, released on November 3, 2011) [20]. As qRT-PCR is easily adapted to 384-well plates, it is possible to carry out high-throughput screening. Here, we describe a procedure for the identification of circulating miRNA biomarkers by qRT-PCR profiling that is composed of four steps: (1) sample collection and preparation; (2) global miRNA profiling using qRT-PCR; (3) data normalization and analysis; (4) selection and validation of miRNA biomarker(s).

### Step 1: Sample collection and preparation

Blood samples can be collected after obtaining the approval of relevant ethics committees and informed consents of donors. All information collected from blood donors, including gender, age, disease grade, symptom, should be recorded. In general, at least tens or hundreds of blood samples should be collected from both pathological and healthy control groups in order to acquire statistically significant data. To reduce costs at the initial screening step, a pooled sample derived from a number of individual specimens (for example, a mixture of 10 to 20 specimens) can be used. Subsequently, the candidate miRNA biomarkers can be further validated with a larger number of samples (> 100) to obtain reliable results [21].

Both serum and plasma are appropriate for the detection of circulating miRNA. However, serum may be preferable to plasma due to the following reasons. First, serum is easier to obtain from clinical sample repositories compared to plasma. Second, plasma is more likely contaminated with platelets and erythrocytes [22]. Finally, some anticoagulants used in plasma collection, such as heparin, inhibit the efficiency of reverse transcription and/or PCR, whereas ethylenediaminetetraacetic acid (EDTA) and citrate are acceptable [23]. It is notable that hemocytolysis during sample collection should be avoided since the products interfere with circulating miRNA quantification. To isolate serum/plasma, blood samples are centrifuged at 3,000 × g for 10 min at 4°C or room temperature. Centrifugation of the serum/plasma can be performed once again at 15,000 × g to remove cell debris [24]. Serum/plasma can be subjected to RNA purification immediately after centrifugation or stored at -80°C, and these procedures should be kept consistent throughout the study to reduce technical variation.

The purification of miRNAs from serum/plasma is difficult because very little amount of miRNAs exist in these samples. In addition, serum/plasma contains numerous inhibitors possibly contaminating the purified RNA that could interfere with subsequent enzymatic reactions. The efficiency of circulating miRNA purification can be monitored by using a heterogenous spike-in RNA, such as synthetic *Caenorhabditis elegans *miRNA (cel-miRNA), which can be added following the mixing of denaturing reagents.

Two specialized types of reagents have been developed for the purification of circulating RNA. The first type is Trizol LS reagent (Invitrogen) or Tri-Reagent BD (Molecular Research Center). After incubation with these denaturing reagents and extraction with phenol/chloroform, inhibiting factors in serum/plasma are removed effectively. Total RNA is then precipitated with ethanol or isopropanol. Higher concentrations of RNA can be obtained using less RNase-free water, which is an advantage of this procedure. However, technical variation usually exists due to the slight loss of RNA during washing and dissolving steps. Moreover, the operation is laborious for processing large numbers of clinical samples mainly due to the precipitation procedure. In contrast, kits using a column-binding strategy, such as miRNeasy (QIAGEN) and mirVana PARIS kits (Ambion), may provide better reproducibility and easier operation. By properly regulating the RNA affinity with ethanol on a solid support such as silica or glass-fiber, small RNAs < 200 nucleotides can bind to the column and then be eluted with RNase-free water. The mirVana PARIS kit is preferred since it applies an equal volume of denaturing reagent with serum/plasma sample, significantly reducing the volume of reagent used per sample and enhancing the efficiency of RNA purification.

Generally, the concentration of total RNA purified from serum/plasma can be measured using specific equipment, for example the NanoDrop spectrophotometer (NanoDrop Technologies), and is usually < 50 ng/uL [25].

### Step 2: Global miRNA profiling using qRT-PCR

Following RNA purification, cDNA synthesis and subsequent miRNA profiling can be carried out by qRT-PCR. Despite remarkable sensitivity and specificity of qRT-PCR method, there are challenges for using this method to analyze miRNA profiles due to the following reasons: (1) miRNAs are too short to provide enough sequence for primer design; (2) many miRNAs are highly conserved in sequence; (3) there are multiple forms of miRNAs, including primary transcript (pri-miRNA), miRNA precursor (pre-miRNA), and mature miRNA, therefore, it requires outstanding specificity to recognize the mature miRNAs. At present, there are two qRT-PCR methods for miRNA expression analyses, the stem-loop method and the poly(A) method.

**Figure 1:**
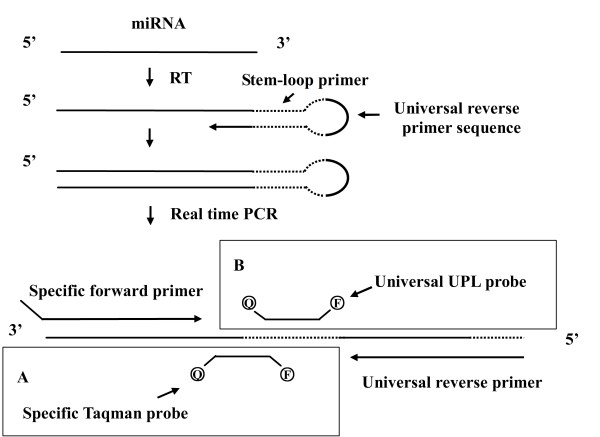
**Schematic representation of the stem-loop method for the study of miRNA expression**. The reverse transcription of miRNA can be performed using a stem-loop RT primer that contains a universal reverse primer sequence on the loop and several specific bases at 3' end that are complementary to the 3'end of mature miRNA. The cDNA is amplified with specific forward and universal reverse primers. The amplification products can be detected by either (A) specific Taqman probe or (B) universal UPL (Universal ProbeLibrary) probe.

The stem-loop method that uses an elaborately designed stem-loop reverse transcription (RT) primer for synthesizing the cDNA of miRNAs, is the most extensively used approach for miRNA quantification (Table 1) [12,18,19,26]. The stem-loop RT primer is an oligonucleotide that forms a stem-loop structure containing a universal reverse primer sequence on the loop and several (usually six) specific bases at 3' end that are complementary to the 3'end of specific mature miRNA (Figure 1) [12]. After RT, the cDNA of miRNA can be quantitatively amplified with specific forward and universal reverse primers. The stem-loop method is able to discriminate mature miRNAs from genomic DNA, pri-miRNA and pre-miRNA, presumably due to the effect of base stacking and spatial restriction of the stem-loop RT primer [12]. In addition, a dual-labeled hydrolytic Taqman probe that is specific for each miRNA is used to ensure the high specificity of miRNA detection (Figure 1A).

**Table 1:** Summary of commercialized qRT-PCR methods for the detection of miRNAs

Method	Forward primer	Reverse primer	Detection method	References
Stem-loop	specific	universal	Specific probe	[54]
Stem-loop	specific	universal	LNA UPL probe	[32]
Poly(A)	specific	universal	SYBR	[55-57]
Poly(A)	LNA specific	LNA specific	SYBR	[58]

LNA: locked nucleic acid; UPL: Universal probe library; SYBR: SYBR Green I.

Initially, individual stem-loop RT primers were used for miRNA detection. Later, pooled stem-loop RT primers were applied for cDNA synthesis of hundreds of miRNAs simultaneously, facilitating high-throughput miRNA profiling [18,19,26]. For instance, using Megaplex Pools (Applied Biosystems) to detect hundreds of miRNAs on 384-well plates with a compatible thermal cycler, such as the Applied Biosystems 7900 HT, many studies identified a number of promising biomarkers associated with cancers and many other diseases [24,25,27-30] (Table 2). However, since the Taqman probes designed for this method are distinct for each miRNA, they are likely to be costly for global miRNAs profiling [14]. Furthermore, the preamplification step in the stem-loop method might cause bias in the miRNA quantification [19]. To overcome the disadvantage of high cost while retaining the high specificity of the stem-loop method, some researchers used a probe from the Universal ProbeLibrary (UPL) for miRNA expression studies (Figure 1B) [31,32]. The UPL probe is a class of probes produced by Roche Diagnostics, each of which contains 8 to 9 nucleotides with some nucleotides modified by a locked nucleic acid (LNA) technique. By integrating the binding sequence of an elaborately selected UPL probe into the conventional stem-loop RT primer, the UPL probe can be used with the comparable specificity to the Taqman probe, resulting in a single probe for the economical detection of various miRNAs [31].

**Table 2:** Summary of circulating miRNA biomarkers identified through qRT-PCR profiling

	Step 1			Step 2	Step 3	Step 4				
					
Disease	**No**. Pa/Nor	Type	Kit	Method	Normalizer	**No**. Pa/Nor	Marker	Function	Statistical analysis	References
ovarian cancer	9/4	S	TR	AB	U44 U48	19/11	miR-21 miR-92 miR-93	Severity	Mann-Whitney	[27]
prostate cancer	21/ N/A	S	TR	AB	cel-miR-39 cel-miR-54 cel-miR-238	113/ N/A	miR-375 miR-141	Severity Tissue	Limma analysis ANOVA	[28]
colorectal cancer	5/5	P	Tz	SBI	U6	115/70	miR-17 -3p miR-92	Severity Tissue Prognosis	Mann-Whitney Wilcoxon the χ^2 ^test Kruskal-Wallis ROC	[21]
HBV infected	51/12	S	mir	AB	U6	51/12	miR-122	Severity	Mann-Whitney	[29]
Crohn disease	46/32	S	mir	AB	miR-302a miR-372 miR-302d cel-miR-54 cel-miR-238	46/32	11 miRNAs*	Severity Prognosis	Mann-Whitney Hierarchical- cluster ROC	[30]
liver disease	5/2	S	mir	AB	U6	112/24	miR-885-5p	Comparison	Mann-Whitney Kruskal-Wallis ROC	[24]

HBV: hepatitis B virus; Pa: patients; Nor: normal; N/A: not available; S: serum; P: plasma; TR: Tri-Reagent BD (Molecular Research Center or Sigma); Tz: Trizol LS reagent (Invitrogen); mir: mirVana™ miRNA isolation kit (Ambion); AB: Megaplex™ Pools (Applied Biosystems); SBI: QuantiMir™ (System Biosciences); Severity: correlation of miRNAs expression level with disease severity; Prognosis: correlation of miRNAs expression level and the treatment of disease; Tissue: correlation of miRNAs expression level in serum with that in tissue; Comparison: comparison between newly identified miRNA biomarkers and conventional biomarkers; ROC: Receiver operating characteristic curve; ANOVA: Analysis of variance.

*: miR-16, miR-195, miR-106a, miR-20a, miR-30e, miR-140, miR-484, miR-93, miR-192, miR-21, let-7b.

**Figure 2:**
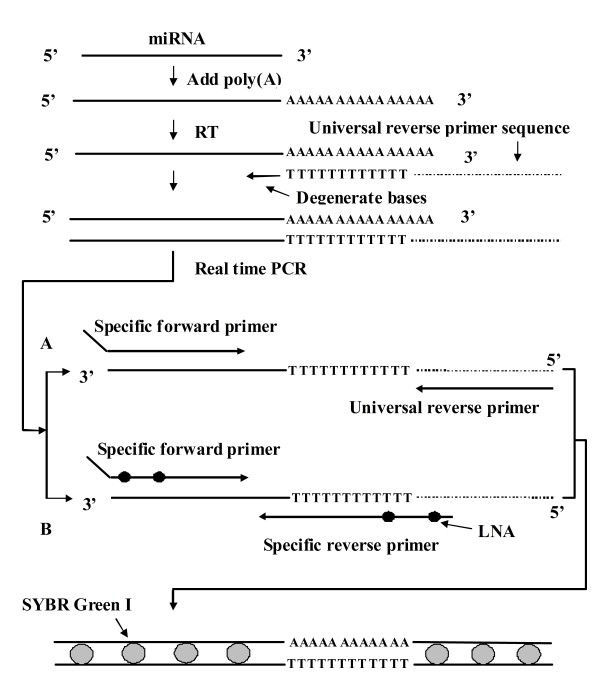
**Schematic representation of the poly(A) method used to study miRNA expression**. After polyadenylation, the reverse transcription of miRNA is carried out using a universal RT primer that contains degenerate nucleotides at 3' end followed by an oligo(dT) and universal reverse primer sequence. The cDNA is amplified with either (A) specific forward and universal reverse primers or (B) LNA-integrated specific forward and specific reverse primers. The amplification products are detected by SYBR Green I.

The poly(A) method is another means used for miRNA expression analysis [14,33,34]. In this method, a poly(A) tail is added to the 3' end of each mature miRNA done by poly(A) polymerase. Tailed miRNAs are then subjected to RT using a universal RT primer containing 2 to 3 degenerate nucleotides at 3' end followed by an oligo(dT) and universal reverse primer sequence (Figure 2). The synthesized cDNA is amplified with specific forward and universal reverse primers (Figure 2A). Instead of using a hydrolytic probe, SYBR Green I is used to quantitatively detect the amplified products and provides a more cost-effective detection method compared with the stem-loop/Taqman probe method. However, all polyadenylated RNAs are possibly recognized by the oligo(dT) RT primer. In addition, since SYBR Green I dye could bind to all double-strand DNA including amplification products of target miRNA, contaminant genomic DNA, and primer-dimers, the poly(A) method is not as specific as the stem-loop method. Nevertheless, the specificity of the poly(A) method can be monitored by observing the melting curve (or dissociation curve).

Many companies, such as SBI (System Biosciences), SAB (SABiosciences), and Invitrogen, have developed kits using the poly(A) method for miRNA profiling (Table 1), due to its cost-effective advantage. For example, circulating miRNA profiling has been performed on plasma collected from patients with colorectal cancer by using the QuantiMir kit (SBI), indicating miR-17-3p and miR-92 as potential biomarkers [21] (Table 2). A modified poly(A) method, miRCURY platform, has been developed recently (Figure 2B, Table 1) by using enhanced LNA-specific forward and reverse primers instead of a universal reverse primer during amplification, achieving superior sensitivity and linearity [35].

Generally, the stem-loop method has been coupled with hydrolytic probes and the poly(A) method with SYBR green I detection; however, these combinations are flexible. For example, SYBR can be used in the stem-loop method [31], and the UPL probe in the poly(A) method [32]. Moreover, the LNA base can be replaced by an extra artificial tail at the 5' end of specific forward/reverse primers in the poly(A) method, achieving considerably higher efficiency than with LNA primers [36]. Overall, both the stem-loop and poly(A) methods as well as their derivatives can be used in circulating miRNA profiling. Currently, miRNA kits from Applied Biosystems that use stem-loop RT primers and specific Taqman probes have the greatest popularity for clinical miRNA evaluation.

Several controls are required for the analysis of circulating miRNAs. A no-template control (NTC) can detect the presence of primer-dimers and other non-specific amplification products. In addition, a no-RT control (NRC) would monitor contamination of the sample with genomic DNA. An inter-run calibrator (IRC), usually using a DNA fragment, is a positive control to calibrate the deviation between qRT-PCR runs/plates. It is of great importance to use an IRC in a profiling assay when various samples are detected in different qRT-PCR runs/plates. Reference controls including spike-in RNA controls are essential for data normalization, as discussed below.

### Step 3: Data normalization and analysis

Data normalization is another major challenge for the analysis of circulating miRNA because there are no verified housekeeping genes existing in serum/plasma that can be used for normalization. To date, three normalization strategies have been proposed for circulating miRNA quantification.

The first strategy uses a stably expressed gene as a reference control according to the existing data, such as miR-16 [37], small nuclear/nucleolar RNAs RNU6 [21,29], RNU44 and RNU48 [38]. Although these reference genes constitute the best possible normalization controls, they cannot ensure constant expression under all experimental conditions [38,39]. Spiked-in RNAs, such as cel-miR-39, cel-miR-54, and cel-miR-238, are a class of heterogenous RNA that can not only monitor the efficiency of RNA purification and RT, but also be used as normalization controls [3,28,30]. By adding the same amount of spiked-in RNAs with an equal volume of serum/plasma, a stable reference control is obtained. However, using an equal mass of circulating total RNA to combine with Spike-in RNAs may result in better accuracy for normalization.

The second strategy of data normalization is to identify suitable normalizers for each study through the systematic evaluation of the expression level of a set of housekeeping genes. Several housekeeping genes are first selected and subjected to qRT-PCR assay using a number of samples. An algorithm named geNorm is then used to calculate the ratio of expression level of one housekeeping gene to another [38]; the algorithm assumes that this ratio is constant across all samples. An M-value is defined as the average standard deviation of the ratio of the pairwise housekeeping genes. The gene with the lowest M-value is determined to have the most stable expression level. A good combination of stably expressed housekeeping genes can be recognized by geNorm, and the geometric average can be used as a normalization factor for qRT-PCR data analysis.

The geNorm program was first used to identify appropriate reference controls for 13 types of human tissue [38]. Later, many studies used this algorithm to successfully identify reliable reference controls for miRNA quantification. For example, let-7a and miR-16 were identified as reference controls for breast cancer [40]. Similarly, miR-191 and miR-103 were chosen as reference controls for lung cancer and 13 distinct human solid tissues [41], as well as circulating miR-22*, miR-26a and miR-221 for hepatitis B virus-infected serum samples [25]. Normfinder is another algorithm used for identification of suitable normalizers [42]. The geNorm and Normfinder program have been integrated in qRT-PCR data analysis software qBaseplus [43,44] or GenEx [45].

It is important to identify a set of miRNAs as reference controls; however, this procedure is labor-intensive because of the numerous miRNAs in a number of samples that need to be measured. Additionally, there may be few relatively stable miRNAs existing in serum/plasma that can be chosen for conducting such a systematic evaluation, which limits its use in circulating miRNA quantification studies.

The third strategy is the newly reported global mean normalization method. Instead of using a single or a set of reference control(s), the global mean method uses the average expression level of all miRNAs detected in a sample as a normalization factor [46]. This strategy assumes that the mean expression level of all miRNAs in a sample, from either the healthy control or the patient, is constant when using the same total RNA input. Mestdagh et al. [46] demonstrated that the global mean method was better in reducing technical variation and preserving biological variation than using endogenous small miRNAs, such as nuclear/nucleolar RNAs. This method is very suitable to normalize genome-wide miRNA profiling without the need for selecting a specific reference control. However, data obtained by this method should be viewed cautiously when many miRNAs analyzed show great variability in expression levels in a sample, since the average value in this case will be apparently different. Moreover, the global mean normalization method is not suitable during the biomarker validation (discussed in the next section) because only few candidate miRNAs will be evaluated. The global mean normalization program is integrated in the qBase^plus ^and GenEx analysis software.

As described above, none of the three normalization strategies provides an ideal solution for profiling circulating miRNAs. To assist analyses, the term Cq is the abbreviation of "quantification of cycle" recommended by the Minimum Information for Publication of Quantitative Real-Time PCR Experiments (MIQE), a substitution of the previously used terms of threshold cycle (Ct), crossing point (Cp) and take off point (TOP) [47,48]. This parameter provides an ideal reference point for the analysis of qRT-PCR assays. Here, we propose a comprehensive strategy. First, omit the miRNA(s) with the Cq value difference > 5 (32-fold change) between the healthy control and the patient sample. Second, calculate the global mean of Cq value of the extra miRNAs in each sample (the healthy control or the patient), which is used as a normalization factor (NF). Third, normalize all miRNAs, including those omitted before, with the formula, ΔCq = Cq_miRNA _- Cq_NF_. Finally, calculate the fold-change between the healthy control and patient with the formula, 2^-ΔΔCq ^[49,50]. Those miRNAs with the least fold-changes might also be used as excellent reference controls in the case studied.

### Step 4: Selection and validation of miRNA biomarkers

In this step, large numbers of samples (50 to 200) are separately used for validating the selected miRNAs in the profiling step. Around 10 to 20 miRNAs with the greatest fold-changes can be selected as candidate biomarkers, each of which needs to be measured in all samples using qRT-PCR assays. Using these analyses, only the miRNAs that are detected in all samples can be considered candidate biomarkers. To identify ideal reference controls for normalizing candidate biomarkers, approximately ten miRNAs with the least fold-changes are also detected in all samples. The algorithm used by the geNorm or Normfinder programs can be used to determine the most stable miRNAs that can serve as the normalization controls. The Cq value of each candidate miRNA marker is then normalized and transformed to the relative expression level using the formula 2^-ΔCq ^[24]. A box plot can then be made to present the relative expression level of the same miRNAs found in all normal or patient samples, with the median line showing whether a differentially-expressed miRNA exists between the healthy and the diseased samples [21,24]. The miRNA(s) with the most significant difference in expression can then act as promising circulating biomarkers.

Significant effort need to be performed to identify a miRNA biomarker with clinical value for the following reasons. First, the selected miRNA must be measured on a more extensive scale for the determination of its clinical applicability. Correlation between the expression level of candidate miRNA markers and other clinical factors, such as age, gender, severity of disease, needs to be evaluated. For example, with a constant expression level during different stages of disease, the miRNA may be a good biomarker for the diagnosis of the early-stage disease. However, if the expression level varies during the development of the disease, the miRNA can serve as a useful indicator for disease classification and prognosis [28]. Furthermore, the candidate miRNAs should be evaluated in pathological tissue, as the abnormal miRNA expression level found in the circulation might be derived from the release of deregulated miRNA in the tissue in the form of microvesicles [51] or Argonaute 2 protein complex [52]. This then provides a solid support for the candidate miRNA to be used as a biomarker if the expression level in the circulation is similar to that in the diseased tissue. Further analyses require blood samples obtained from patients of pre- and post-treatment, for example, surgical removal of carcinoma, to determine the utility of the miRNA biomarker for prognosis.

Finally, it is necessary to detect the candidate miRNA in other relevant diseases since many diseases may have a similar miRNA expression pattern. For example, miR-885-5p is elevated in liver-associated diseases encompassing hepatocellular carcinoma, liver cirrhosis, and chronic hepatitis B [24]. In this case, a set of miRNA biomarkers may provide better specificity for the diagnosis of each disease.

For the analyses of potential biomarkers of disease, it is necessary to performed appropriate statistical analyses of data obtained from the qRT-PCR assay. Generally, the Mann-Whitney and Kruskal-Wallis tests are used for the evaluation of the healthy control versus patient data and among different sets of samples, respectively. However, the Wilcoxon test is appropriate for the analysis of samples collected from the same patient over different periods of treatment. The results that display a p-value < 0.05 represent statistically significant changes. The receiver operating characteristic curve (ROC) can further determine whether the candidate miRNA biomarker is sufficiently specific for discriminating certain type of diseased samples from others (Table 2) [21,30].

### Future directions

Although many circulating miRNAs have already been identified as potential biomarkers, analyses including a larger number of clinical samples are needed to validate these selected miRNAs as effective biomarkers. Moreover, the accuracy of circulating miRNA profiling is wholly reliant on the sample type, sample processing and profiling method, and normalization strategy, so that the procedure should be standardized to make the data obtained from different labs comparable. The highest number of circulating miRNA detected in human serum is 368 [28], however it is unknown whether more miRNAs exist in the serum due to the sensitivity limit of current techniques. Given that many miRNAs with a Cq value of > 35 in qRT-PCR assays are frequently excluded in the normalization step, there are presumably more than 368 miRNAs in human serum. Thus, the development of more sensitive and specific techniques to increase the range of detectable miRNA in serum could reveal more potential biomarkers. To date, a great number of studies on circulating miRNA profiles have mainly focused on human specimens. However, miRNA profiling in animal is in its early stages, such as miRNA profiling performed on cow milk using solexa sequencing [53]. Through these analyses, seven promising biomarkers have been identified that can be used to discriminate milk quality. Further application of qRT-PCR in animal miRNA profiling may identify more useful biomarkers for the diagnosis of animal diseases and quality control. Moreover, significant reduction of the operation cost of qRT-PCR will promote its use in animal studies.

## Conclusion

The use of qRT-PCR is by far the most sensitive and specific means for the evaluation of miRNA profiles. Using a four-step operation of sample preparation, profiling, normalization and validation, promising miRNA biomarkers can be elucidated for clinical diagnosis and prognosis. It is possible that the detection of circulating miRNA biomarkers can be included in future routine clinical examinations for the diagnosis of early stages of diseases, such as cancer and cardiovascular disease.

## Competing interests

The authors declare that they have no competing interests.

## Authors' contributions

KK reviewed the literatures, wrote and drafted the manuscript; XP, JL and DG revised and finalized the manuscript. All authors read and approved the final version.

## References

[B1] Bartel DP MicroRNAs: target recognition and regulatory functions Cell 2009 136 215 233 10.1016/j.cell.2009.01.002 19167326 PMC3794896

[B2] Zhang S Chen L Jung EJ Calin GA Targeting microRNAs with small molecules: from dream to reality Clin Pharmacol Ther 2010 87 754 758 10.1038/clpt.2010.46 20428111 PMC3902962

[B3] Mitchell PS Parkin RK Kroh EM Fritz BR Wyman SK Pogosova-Agadjanyan EL Peterson A Noteboom J O'Briant KC Allen A Lin DW Urban N Drescher CW Knudsen BS Stirewalt DL Gentleman R Vessella RL Nelson PS Martin DB Tewari M Circulating microRNAs as stable blood-based markers for cancer detection Proc Natl Acad Sci USA 2008 105 10513 10518 10.1073/pnas.0804549105 18663219 PMC2492472

[B4] Brase JC Wuttig D Kuner R Sultmann H Serum microRNAs as non-invasive biomarkers for cancer Mol Cancer 2010 9 306 315 10.1186/1476-4598-9-306 21110877 PMC3002336

[B5] Yu DC Li QG Ding XW Ding YT Circulating MicroRNAs: Potential Biomarkers for Cancer Int J Mol Sci 2011 12 2055 2063 10.3390/ijms12032055 21673939 PMC3111650

[B6] D'Alessandra Y Devanna P Limana F Straino S Di Carlo A Brambilla PG Rubino M Carena MC Spazzafumo L De Simone M Micheli B Biglioli P Achilli F Martelli F Maggiolini S Marenzi G Pompilio G Capogrossi MC Circulating microRNAs are new and sensitive biomarkers of myocardial infarction Eur Heart J 2010 31 2765 2773 10.1093/eurheartj/ehq167 20534597 PMC2980809

[B7] Dimmeler S Zeiher AM Circulating microRNAs: novel biomarkers for cardiovascular diseases? Eur Heart J 2010 31 2705 2707 10.1093/eurheartj/ehq221 20605798

[B8] Valoczi A Hornyik C Varga N Burgyan J Kauppinen S Havelda Z Sensitive and specific detection of microRNAs by northern blot analysis using LNA-modified oligonucleotide probes Nucleic Acids Res 2004 32 175 183 10.1093/nar/gnh171 PMC545470 15598818

[B9] Lu J Getz G Miska EA Alvarez-Saavedra E Lamb J Peck D Sweet-Cordero A Ebert BL Mak RH Ferrando AA Downing JR Jacks T Horvitz HR Golub TR MicroRNA expression profiles classify human cancers Nature 2005 435 834 838 10.1038/nature03702 15944708

[B10] Fichtlscherer S De Rosa S Fox H Schwietz T Fischer A Liebetrau C Weber M Hamm CW Roxe T Muller-Ardogan M Bonauer A Zeiher AM Dimmeler S Circulating microRNAs in patients with coronary artery disease Circ Res 2010 107 677 684 10.1161/CIRCRESAHA.109.215566 20595655

[B11] Zhao H Shen J Medico L Wang D Ambrosone CB Liu S A pilot study of circulating miRNAs as potential biomarkers of early stage breast cancer PLoS One 2010 5 13735 13747 10.1371/journal.pone.0013735 PMC2966402 21060830

[B12] Chen C Ridzon DA Broomer AJ Zhou Z Lee DH Nguyen JT Barbisin M Xu NL Mahuvakar VR Andersen MR Lao KQ Livak KJ Guegler KJ Real-time quantification of microRNAs by stem-loop RT-PCR Nucleic Acids Res 2005 33 179 188 10.1093/nar/gni178 PMC1292995 16314309

[B13] Yang H Schmuke JJ Flagg LM Roberts JK Allen EM Ivashuta S Gilbertson LA Armstrong TA Christian AT A novel real-time polymerase chain reaction method for high throughput quantification of small regulatory RNAs Plant Biotechnol J 2009 7 621 630 10.1111/j.1467-7652.2009.00429.x 19619184

[B14] Benes V Castoldi M Expression profiling of microRNA using real-time quantitative PCR, how to use it and what is available Methods 2010 50 244 249 10.1016/j.ymeth.2010.01.026 20109550

[B15] Hu Z Chen X Zhao Y Tian T Jin G Shu Y Chen Y Xu L Zen K Zhang C Shen H Serum microRNA signatures identified in a genome-wide serum microRNA expression profiling predict survival of non-small-cell lung cancer J Clin Oncol 2010 28 1721 1726 10.1200/JCO.2009.24.9342 20194856

[B16] Wu Q Lu Z Li H Lu J Guo L Ge Q Next-generation sequencing of microRNAs for breast cancer detection J Biomed Biotechnol 2011 2011 597145 597152 21716661 10.1155/2011/597145 PMC3118289

[B17] Kong W Zhao JJ He L Cheng JQ Strategies for profiling microRNA expression J Cell Physiol 2009 218 22 25 10.1002/jcp.21577 18767038

[B18] Tang F Hajkova P Barton SC Lao K Surani MA MicroRNA expression profiling of single whole embryonic stem cells Nucleic Acids Res 2006 34 9 16 10.1093/nar/gnj009 PMC1351374 16434699

[B19] Mestdagh P Feys T Bernard N Guenther S Chen C Speleman F Vandesompele J High-throughput stem-loop RT-qPCR miRNA expression profiling using minute amounts of input RNA Nucleic Acids Res 2008 36 143 151 10.1093/nar/gkn725 PMC2588502 18940866

[B20] Kozomara A Griffiths-Jones S miRBase: integrating microRNA annotation and deep-sequencing data Nucleic Acids Res 2011 39 152 157 10.1093/nar/gkq1027 PMC3013655 21037258

[B21] Ng EK Chong WW Jin H Lam EK Shin VY Yu J Poon TC Ng SS Sung JJ Differential expression of microRNAs in plasma of patients with colorectal cancer: a potential marker for colorectal cancer screening Gut 2009 58 1375 1381 10.1136/gut.2008.167817 19201770

[B22] McDonald JS Milosevic D Reddi HV Grebe SK Algeciras-Schimnich A Analysis of circulating microRNA: preanalytical and analytical challenges Clin Chem 2011 57 833 840 10.1373/clinchem.2010.157198 21487102

[B23] Kroh EM Parkin RK Mitchell PS Tewari M Analysis of circulating microRNA biomarkers in plasma and serum using quantitative reverse transcription-PCR (qRT-PCR) Methods 2010 50 298 301 10.1016/j.ymeth.2010.01.032 20146939 PMC4186708

[B24] Gui J Tian Y Wen X Zhang W Zhang P Gao J Run W Tian L Jia X Gao Y Serum microRNA characterization identifies miR-885-5p as a potential marker for detecting liver pathologies Clin Sci (Lond) 2011 120 183 193 10.1042/CS20100297 20815808 PMC2990200

[B25] Zhu HT Dong QZ Wang G Zhou HJ Ren N Jia HL Ye QH Qin LX Identification of Suitable Reference Genes for qRT-PCR Analysis of Circulating microRNAs in Hepatitis B Virus-Infected Patients Mol Biotechnol 2012 50 49 56 10.1007/s12033-011-9414-6 21567136

[B26] Lao K Xu NL Sun YA Livak KJ Straus NA Real time PCR profiling of 330 human micro-RNAs Biotechnol J 2007 2 33 35 10.1002/biot.200600119 17124719

[B27] Resnick KE Alder H Hagan JP Richardson DL Croce CM Cohn DE The detection of differentially expressed microRNAs from the serum of ovarian cancer patients using a novel real-time PCR platform Gynecol Oncol 2009 112 55 59 10.1016/j.ygyno.2008.08.036 18954897

[B28] Brase JC Johannes M Schlomm T Falth M Haese A Steuber T Beissbarth T Kuner R Sultmann H Circulating miRNAs are correlated with tumor progression in prostate cancer Int J Cancer 2011 128 608 616 10.1002/ijc.25376 20473869

[B29] Ji F Yang B Peng X Ding H You H Tien P Circulating microRNAs in hepatitis B virus-infected patients J Viral Hepat 2011 18 242 251 10.1111/j.1365-2893.2011.01443.x 21692939

[B30] Zahm AM Thayu M Hand NJ Horner A Leonard MB Friedman JR Circulating microRNA is a biomarker of pediatric Crohn disease J Pediatr Gastroenterol Nutr 2011 53 26 33 10.1097/MPG.0b013e31822200cc 21546856 PMC3807879

[B31] Varkonyi-Gasic E Wu R Wood M Walton EF Hellens RP Protocol: a highly sensitive RT-PCR method for detection and quantification of microRNAs Plant Methods 2007 3 12 24 10.1186/1746-4811-3-12 17931426 PMC2225395

[B32] Reichenstein I Aizenberg N Goshen M Bentwich Z Avni YS A novel qPCR assay for viral encoded microRNAs J Virol Methods 2010 163 323 328 10.1016/j.jviromet.2009.10.018 19879298

[B33] Shi R Chiang VL Facile means for quantifying microRNA expression by real-time PCR Biotechniques 2005 39 519 525 10.2144/000112010 16235564

[B34] Zhang Y Li M Wang H Fisher WE Lin PH Yao Q Chen C Profiling of 95 microRNAs in pancreatic cancer cell lines and surgical specimens by real-time PCR analysis World J Surg 2009 33 698 709 10.1007/s00268-008-9833-0 19030927 PMC2933040

[B35] Jensen SG Lamy P Rasmussen MH Ostenfeld MS Dyrskjot L Orntoft TF Andersen CL Evaluation of two commercial global miRNA expression profiling platforms for detection of less abundant miRNAs BMC Genomics 2011 12 435 461 10.1186/1471-2164-12-435 21867561 PMC3184117

[B36] Balcells I Cirera S Busk PK Specific and sensitive quantitative RT-PCR of miRNAs with DNA primers BMC Biotechnol 2011 11 70 81 10.1186/1472-6750-11-70 21702990 PMC3135530

[B37] Wei J Gao W Zhu CJ Liu YQ Mei Z Cheng T Shu YQ Identification of plasma microRNA-21 as a biomarker for early detection and chemosensitivity of non-small cell lung cancer Chin J Cancer 2011 30 407 414 10.5732/cjc.010.10522 21627863 PMC4013415

[B38] Vandesompele J De Preter K Pattyn F Poppe B Van Roy N De Paepe A Speleman F Accurate normalization of real-time quantitative RT-PCR data by geometric averaging of multiple internal control genes Genome Biol 2002 3 34 46 10.1186/gb-2002-3-7-research0034 PMC126239 12184808

[B39] Wu F Zhang S Dassopoulos T Harris ML Bayless TM Meltzer SJ Brant SR Kwon JH Identification of microRNAs associated with ileal and colonic Crohn's disease Inflamm Bowel Dis 2010 16 1729 1738 10.1002/ibd.21267 20848482 PMC2946509

[B40] Davoren PA McNeill RE Lowery AJ Kerin MJ Miller N Identification of suitable endogenous control genes for microRNA gene expression analysis in human breast cancer BMC Mol Biol 2008 9 76 87 10.1186/1471-2199-9-76 18718003 PMC2533012

[B41] Peltier HJ Latham GJ Normalization of microRNA expression levels in quantitative RT-PCR assays: identification of suitable reference RNA targets in normal and cancerous human solid tissues RNA 2008 14 844 852 10.1261/rna.939908 18375788 PMC2327352

[B42] Andersen CL Jensen JL Orntoft TF Normalization of real-time quantitative reverse transcription-PCR data: a model-based variance estimation approach to identify genes suited for normalization, applied to bladder and colon cancer data sets Cancer Res 2004 64 5245 5250 10.1158/0008-5472.CAN-04-0496 15289330

[B43] Hellemans J Mortier G De Paepe A Speleman F Vandesompele J qBase relative quantification framework and software for management and automated analysis of real-time quantitative PCR data Genome Biol 2007 8 19 33 10.1186/gb-2007-8-2-r19 PMC1852402 17291332

[B44] The qRT-PCR data analysis software qBaseplus http://www.biogazelle.com/products

[B45] The qRT-PCR data analysis software GenEx http://www.multid.se/

[B46] Mestdagh P Van Vlierberghe P De Weer A Muth D Westermann F Speleman F Vandesompele J A novel and universal method for microRNA RT-qPCR data normalization Genome Biol 2009 10 64 74 10.1186/gb-2009-10-6-r64 PMC2718498 19531210

[B47] Bustin SA Benes V Garson JA Hellemans J Huggett J Kubista M Mueller R Nolan T Pfaffl MW Shipley GL Vandesompele J Wittwer CT The MIQE guidelines: minimum information for publication of quantitative real-time PCR experiments Clin Chem 2009 55 611 622 10.1373/clinchem.2008.112797 19246619

[B48] Bustin SA Why the need for qPCR publication guidelines?--The case for MIQE Methods 2010 50 217 226 10.1016/j.ymeth.2009.12.006 20025972

[B49] Schmittgen TD Livak KJ Analyzing real-time PCR data by the comparative C(T) method Nat Protoc 2008 3 1101 1108 10.1038/nprot.2008.73 18546601

[B50] Livak KJ Schmittgen TD Analysis of relative gene expression data using real-time quantitative PCR and the 2(-Delta Delta C(T)) Method Methods 2001 25 402 408 10.1006/meth.2001.1262 11846609

[B51] Hunter MP Ismail N Zhang X Aguda BD Lee EJ Yu L Xiao T Schafer J Lee ML Schmittgen TD Nana-Sinkam SP Jarjoura D Marsh CB Detection of microRNA expression in human peripheral blood microvesicles PLoS One 2008 3 3694 3705 10.1371/journal.pone.0003694 PMC2577891 19002258

[B52] Arroyo JD Chevillet JR Kroh EM Ruf IK Pritchard CC Gibson DF Mitchell PS Bennett CF Pogosova-Agadjanyan EL Stirewalt DL Tait JF Tewari M Argonaute2 complexes carry a population of circulating microRNAs independent of vesicles in human plasma Proc Natl Acad Sci USA 2011 108 5003 5008 10.1073/pnas.1019055108 21383194 PMC3064324

[B53] Chen X Gao C Li H Huang L Sun Q Dong Y Tian C Gao S Dong H Guan D Hu X Zhao S Li L Zhu L Yan Q Zhang J Zen K Zhang CY Identification and characterization of microRNAs in raw milk during different periods of lactation, commercial fluid, and powdered milk products Cell Res 2010 20 1128 1137 10.1038/cr.2010.80 20548333

[B54] Applied Biosystems Megaplex™ Pools http://www.appliedbiosystems.com/absite/us/en/home.html

[B55] System Biosciences QuantiMir™ http://www.systembio.com/

[B56] SABiosciences RT^2 ^miRNA PCR Array http://www.sabiosciences.com/

[B57] Invitrogen NCode EXPRESS SYBR GreenER miRNA qRT-PCR Kit http://www.invitrogen.com/site/us/en/home.html

[B58] Exiqon miRCURY LNA™ Universal RT microRNA PCR http://www.exiqon.com/

